# A Glycoengineered Interferon-β Mutein (R27T) Generates Prolonged Signaling by an Altered Receptor-Binding Kinetics

**DOI:** 10.3389/fphar.2018.01568

**Published:** 2019-01-24

**Authors:** Saehyung Lee, Woo Sung Son, Ho Bin Yang, Nirmal Rajasekaran, Sung-Su Kim, Sungyoul Hong, Joon-Seok Choi, Jun Young Choi, Kyoung Song, Young Kee Shin

**Affiliations:** ^1^Laboratory of Molecular Pathology and Cancer Genomics, Research Institute of Pharmaceutical Sciences and College of Pharmacy, Seoul National University, Seoul, South Korea; ^2^Department of Pharmacy, College of Pharmacy, CHA University, Pocheon, South Korea; ^3^The Center for Companion Diagnostics, LOGONE Bio Convergence Research Foundation, Seoul, South Korea; ^4^College of Pharmacy, Daegu Catholic University, Gyeongsan, South Korea; ^5^R&D Center, ABION Inc., Seoul, South Korea; ^6^Molecular Medicine and Biopharmaceutical Sciences, Graduate School of Convergence Science and Technology, Seoul National University, Suwon, South Korea

**Keywords:** interferon-beta, receptor-binding kinetics, Fc-fusion protein, surface plasmon resonance, glycoengineering, multiple sclerosis

## Abstract

The glycoengineering approach is used to improve biophysical properties of protein-based drugs, but its direct impact on binding affinity and kinetic properties for the glycoengineered protein and its binding partner interaction is unclear. Type I interferon (IFN) receptors, composed of IFNAR1 and IFNAR2, have different binding strengths, and sequentially bind to IFN in the dominant direction, leading to activation of signals and induces a variety of biological effects. Here, we evaluated receptor-binding kinetics for each state of binary and ternary complex formation between recombinant human IFN-β-1a and the glycoengineered IFN-β mutein (R27T) using the heterodimeric Fc-fusion technology, and compared biological responses between them. Our results have provided evidence that the additional glycan of R27T, located at the binding interface of IFNAR2, destabilizes the interaction with IFNAR2 via steric hindrance, and simultaneously enhances the interaction with IFNAR1 by restricting the conformational freedom of R27T. Consequentially, altered receptor-binding kinetics of R27T in the ternary complex formation led to a substantial increase in strength and duration of biological responses such as prolonged signal activation and gene expression, contributing to enhanced anti-proliferative activity. In conclusion, our findings reveal *N*-glycan at residue 25 of R27T is a crucial regulator of receptor-binding kinetics that changes biological activities such as long-lasting activation. Thus, we believe that R27T may be clinically beneficial for patients with multiple sclerosis.

## Introduction

Glycosylation is the most common post-translational process in which carbohydrate moieties are attached enzymatically to protein residues to provide the stability of structure and dynamics (Mitra et al., 2006; Hanson et al., 2009; Aebi et al., 2010; Lee et al., 2015). In protein drug development, the introduction of carbohydrate moieties helps impact the modification of biophysical and pharmacological properties and overcome the inherent limitations of protein-based drugs. Oligosaccharide moieties can increase the overall clearance rate in the serum by altering pharmacokinetic properties (Sinclair and Elliott, 2005), decrease immunogenicity by masking immunogenic epitopes (Lis and Sharon, 1993) and minimize protein aggregation (Høiberg-Nielsen et al., 2006). In addition, recent studies reveal that the glycosylation affects protein–protein interactions (PPIs) leading to changes in biological responses including signal transduction (Nguyen et al., 2003; Bousfield et al., 2004). For example, the N-linked glycan at residue 196 of the interleukin 5 receptor alpha subunit contributes to the ability of ligand binding and alters its biological activity (Ishino et al., 2011). Since glycosylation is highly heterogenic, owing to the factors such as attachment site and glycoforms, the understanding of how a particular site of glycosylation affects biological activity is not clear (Liu, 1992; Davis, 2002).

Type I interferons (IFNs) are key multifunctional mediators of anti-viral, growth-inhibitory, and immune modulatory effects that act directly or indirectly in biological processes (Brassard et al., 2002; Brierley and Fish, 2002) and are useful for treating neoplastic, viral, and autoimmune diseases—in particular, IFN-β is effective in patients with relapsing-remitting multiple sclerosis (IMSS Group, 1993; McHutchison et al., 1998; Cascinelli et al., 2001). Type I IFNs are divided into IFN-α (13 subtypes), IFN-β, IFN-ε, IFN-κ, and IFN-ω (Pestka et al., 1987; Platanias, 2005). Type I IFNs are selectively recognized by common cell surface receptors composed of IFNAR1 and IFNAR2. These receptors belong to the class II cytokine receptor family (Heldin, 1995), which initiate strikingly different biological effects via a two-step binding mechanism (Subramaniam et al., 1995; Russell-Harde et al., 1999; Jaitin et al., 2006). In the canonical pathway of type I IFNs, binding to IFNAR2 results in the binary complex formation, and subsequent formation of a ternary complex with IFNAR1, thereby activating the receptor-associated protein tyrosine kinases Janus kinase 1 and tyrosine kinase 2. Thereafter, signal transducers and activators of transcription (STAT) proteins, especially STAT1 and STAT2, are phosphorylated to form a dimer, followed by binding of IFN-regulatory factor 9 to form a heterotrimer (Levy David and Darnell, 2002; Stark and Darnell, 2012). This complex, called interferon-stimulated gene factor 3 (Fu et al., 1990) translocates into the nucleus, where it induces expression of hundreds of interferon-stimulated genes (ISGs) via their IFN-stimulated response elements (ISREs) (Ivashkiv and Donlin, 2014) and thereby regulates a diverse range of biological processes (Darnell et al., 1994; Au-Yeung et al., 2013). A number of studies reveal a direct correlation between biological activity of type I IFN and its receptor-binding properties, suggesting a key role for receptor assembly and dynamics in signaling specificity (Jaks et al., 2007; Lavoie et al., 2011; Piehler et al., 2012; Schreiber and Piehler, 2015). For example, receptor-binding studies of type I IFN demonstrated that IFN-β elicits stronger anti-proliferative and anti-viral effects than other type I IFNs including IFN-α, due to its high affinity for IFNAR2 and IFNAR1 (Roisman et al., 2005; Kalie et al., 2007). In addition, an IFN-α mutant (YNS) and its engineered forms with increased binding affinity for type I IFN receptors exhibited not only higher anti-proliferative activity than IFN-β but also had therapeutic effect in an animal model of multiple sclerosis (Harari et al., 2014). Whereas, another IFN-α2 mutant binds tightly to IFNAR2 but not to IFNAR1, resulting in an IFNAR1 antagonist with partial anti-viral activity and no anti-proliferative activity (Pan et al., 2008). These studies have proven to be beneficial in the development of novel therapeutic strategies (Copeland et al., 2006).

We have developed a hyper-glycosylated IFN-β mutein, R27T, in which a glycan is located at the N25 residue, and we found that this modification changes the receptor-binding kinetics. The main purpose of this study is to gain an insight into the role of this additional N-linked glycan in receptor-ligand binding kinetics and their cellular responses. In the present study, we hypothesized that site-specific glycosylation of R27T affects receptor-binding kinetics during ligand-induced assembly of the type I IFN receptor, resulting in changes in the cellular activities. Herein, we assessed the binding kinetics of IFN-β-1a and R27T with each receptor, as well as in the formation of ternary complexes, using heterodimeric Fc-fusion technology.

## Materials and Methods

### IFN-β Preparation

R27T samples, which were purified according to published methods, were produced by AbionBio Inc. (Seoul, South Korea) using a Chinese hamster ovary cell line (Song et al., 2014). An additional glycosylation was introduced at residue 25 by the site-directed mutagenesis of Arg27 to Thr. The commercial IFN-β-1a product Rebif^®^(Hesse, Germany) was used as a control. For SPR analysis, both samples were concentrated using an Amicon Ultra 10 kDa cut-off centrifugation filter device (Millipore Inc., Bedford, MA, United States), and buffer-exchanged into Dulbecco’s phosphate-buffered saline (DPBS/modified; Hyclone Inc., Logan, UT, United States). Protein concentrations were determined by measuring the absorbance at 280 nm for R27T (extinction coefficient = 1.615 mL/mg.cm) and at 290 nm for IFN-β-1a (extinction coefficient = 1.71 mL/mg.cm).

### Gene Construction, Protein Expression and Purification, and Complex Formation

All recombinant Fc-fusion proteins used in this study were expressed as C-terminally Fc-tagged forms (encoded by IGHG1 and P01857; residues 100-330) using a polypeptide linker (IEGRMD) following cloning into a modified version of the pOptiVec-TOPO plasmid (pFC; Invitrogen) containing the optimal secretion signal sequence for Expi293 transient expression (Barash et al., 2002). The extracellular domain (ECD) of human IFNAR1 (P17181, residues 28-436) and IFNAR2 (P48551, residues 27-243) was encoded between the optimal signal and linker sequences. All genes were synthesized using GeneArt Strings DNA Fragments (Thermo Fisher Scientific, Waltham, MA, United States) and inserted into pOptiVec-TOPO using *Nhe*I and *Xho*I restriction enzyme sites. According to the ‘EW-RVT’ strategy, constructs for mutants K360E and K409W of pFC_EW_ and pIFNAR1-FC_EW_, and Q347R, D399V and F405T of pFC_RV T_, pIFNAR1-FC_RV T_, and pIFNAR2-FC_RV T_, respectively, were prepared to replace a region of the CH3 domain with the IGHG1 sequence using site-directed mutagenesis. Fc-fusion proteins were produced in transiently co-transfected Expi293 cells (Invitrogen) according to the manufacturer’s protocol using pIFNAR1-FC_EW_/pIFNAR2-FC_RV T_, pIFNAR1-FC_RV T_/pFC_EW_, and pIFNAR2-FC_RV T_/pFC_EW_ plasmid sets, and purified from the expression media using MabSelect SuRe (GE Healthcare, Uppsala, Sweden). All chromatographic experiments were performed on an AKTA Avant chromatography system (GE Healthcare). Pooled fractions were dialyzed against 20 mM sodium phosphate pH 7.4 and 150 mM NaCl, and concentrated using an Amicon Ultracel-50K centrifugal concentrator (Merck Millipore). Concentrated samples were applied to a HiLoad 16/600 Superdex 200 pg HR gel filtration chromatography column (GE Healthcare) equilibrated with DPBS/modified (Hyclone Inc.). Eluted fractions were pooled and concentrated to 2 mL, and concentrations were determined by a turbidometric method using Cedex bio HT (Roche). Purified AR1/2Fc protein (2 μM) was mixed with 20 μM R27T or IFN-β-1a in DPBS buffer (pH 7.4) and incubated for 1 h at 37°C with rotation in a shaking incubator (TAITEC, Saitama, Japan). Samples were confirmed by PAGE analysis under native (7.5% Mini-PROTEIN TGX gel, Bio-Rad Laboratories) and reducing (Any Kd Mini-PROTEIN TGX gel, Bio-Rad Laboratories) conditions and stained with Coomassie Brilliant Blue R-250 (Biosolution Co., Seoul, South Korea), and each band was analyzed using Image Lab software (Bio-Rad Laboratories).

### SPR Experiments

Ligand–receptor interaction kinetics and affinity measurements were performed at 25°C using a BIAcore T200 SPR instrument (GE Healthcare) with DPBS-T (DPBS/modified and 0.005% Tween 20) as the running buffer. Approximately 5,000 resonance units (RUs) of anti-human IgG Fcγ fragment (Cat no. 109-005-008; Jackson ImmunoResearch Laboratories Inc., West Grove, PA, United States) was immobilized on a CD5 sensor chip using default parameters in the BIAcore T200 control software. Fc-fusion proteins (AR1Fc, AR2Fc, or AR1/2Fc) were reversibly captured at a loading of ∼80-100 RU onto the anti-human Fc surface, and a non-captured flow cell acted as a blank control. R27T and IFN-β-1a were prepared at various concentrations in DPBS-T running buffer, and twofold serially diluted samples were injected at a constant flow rate of 30 μL/min to determine association and dissociation rate constants. Sensorgram data were processed and fitted using BIA evaluation software (BIAcore, Uppsala, Sweden).

### Cell Culture and Western Blot Analysis

Daudi, Ramos, and Jurkat cells were purchased from the Korean Cell Line Bank (KCLB, Seoul, South Korea). All cell lines were cultured in RPMI1640 medium supplemented with 10% fetal bovine serum, 100 units/mL penicillin, and 100 μg/mL streptomycin. All cell culture media and supplements were purchased from Hyclone Inc. Cells were grown at 37°C with 5% CO_2_ in a humidified atmosphere incubator. Cells (5 × 10^5^ cells/well) were seeded in 12-well plates and treated with the indicated concentrations of R27T, IFN-β-1a (Rebif), and IFN-β-1a (PBL Interferon Source). Treated cells were harvested at different time points and lysed in RIPA buffer (Biosesang Inc., Seongnam, South Korea) containing protease inhibitor cocktail and phosphatase inhibitor (Roche Diagnostics GmbH, Mannheim, Germany). Samples were separated on a 10% SDS-PAGE gel and transferred onto a polyvinylidene fluoride (PVDF) membrane (Millipore). Antibodies used for western blotting were as follows: rabbit anti-STAT1 (1:2000, 06-501, Merck Millipore, Billerica, MA, United States), rabbit anti-pSTAT1 Tyr701 (1:1000, 9167, Cell Signaling Technology, Inc., Beverly, MA, United States), mouse anti-β-actin (1:3000, sc-47778, Santa Cruz Biotechnology, Dallas, TX, United States), horseradish peroxidase-conjugated goat anti-rabbit IgG (1:3000, 31460, Invitrogen, Carlsbad, CA, United States), and horseradish peroxidase-conjugated goat anti-mouse IgG (1:3000, G21040, Invitrogen). Chemiluminescent signals were detected using the ECL Clarity Western ECL Substrate (Bio-Rad Laboratories, Hercules, CA, United States).

### RNA Preparation and Quantitative Real-Time Polymerase Chain Reaction (qRT-PCR) Analysis

Total RNA was isolated using a Hybrid-R RNA Purification Kit (GeneAll, Seoul, South Korea), and cDNA was synthesized from 2 μg of total RNA using a Transcriptor First-strand cDNA Synthesis Kit (Roche) according to the manufacturer’s protocol. For real-time PCR, all genes were evaluated with SYBR Green Premix Ex Taq (TaKaRa Bio Inc., Dalian, China) in duplicate on a 7500 Fast Real-Time PCR System (Applied Biosystems, Darmstadt, Germany). Sequences of primer pairs used for RT-PCR analysis are listed in Supplementary Table S1. The expression levels of the target genes relative to that of GAPDH were determined using the comparative CT method (fold change in target genes relative to untreated sample = 2^-ΔΔCT^).

### Cell Viability and Competitive Binding Assays

The *in vitro* cell viability was determined using an EZ-Cytox colorimetric cell viability assay kit according to the product instructions (Daeil Lab Service Co. Ltd., Seoul, South Korea). In brief, Daudi cells were seeded at a density of 3 × 10^3^ cells/well containing fivefold serially diluted IFN-βs (R27T or IFN-β-1a) in a 96-well plate and incubated for 72 h. Reagents were added, and samples were further incubated for 3 h. The optical density was measured at a wavelength of 430 nm using a Tecan GENios Multiplate Reader (Tecan, Raleigh, NC, United States). IC_50_ values were calculated by non-linear regression analysis using GraphPad Prism 7 (GraphPad Software, San Diego, CA, United States). For the competitive binding assay, Daudi cells were incubated for 72 h with either 1 nM of IFN-βs alone (mock) or with Fc-fusion proteins, and the IC50 values were determined on the basis of the cell viability assay results. Thereafter, the value of the IC50 fold change was calculated by dividing it by the mock value.

### Molecular Docking

Molecular models of R27T were built based on the crystal structure of IFN-β (PDB ID: 1AU1). Mutation of arginine to threonine at residue 27 and N-linked glycosylation of 1AU1 were performed using UCSF Chimera (Pettersen et al., 2004) and GLYCAM (GLYCAM Web^1^, Complex Carbohydrate Research Center, University of Georgia, Athens, GA, United States) (Woods et al., 1995; Kirschner et al., 2008). To generate the properly glycosylated structure, the angles of the sidechain of the asparagine residue at position 25 were set to 59.7° (chi1) and 50.0° (chi2) using the Dunbrack rotamer library implemented in UCSF Chimera. The initial structure of the R27T/IFNAR complex was generated via structural alignment using the model of the IFN-α2/IFNAR ternary complex, which was previously determined by X-ray crystallography (PDB ID: 3SE4). Sub-domain 4 of IFNAR1, which is missing from the template structure of the complex, was added to the complex model based on another IFNAR1 structure (PDB ID: 3WCY). The structure of the complex was minimized after cleaning up and the addition of hydrogen using YASARA (Krieger et al., 2002; Krieger and Vriend, 2014; Land and Humble, 2018). All structures were presented using UCSF Chimera and YASARA.

### Statistical Analysis

All values are presented as means ± standard deviation (SD). Where indicated, significance was analyzed using one or two-way analysis of variance (ANOVA) with appropriate *post hoc* analysis for multiple groups, or student’s unpaired two-tailed *t*-tests. Significance was defined at ^∗^*p* < 0.05, ^∗∗^*p* < 0.01, ^∗∗∗^*p* < 0.001 using GraphPad Prism 7.0 software.

## Results

### Design, Expression of Heterodimeric Type I IFN Receptor Fc-Fusion Proteins

**FIGURE 1:**
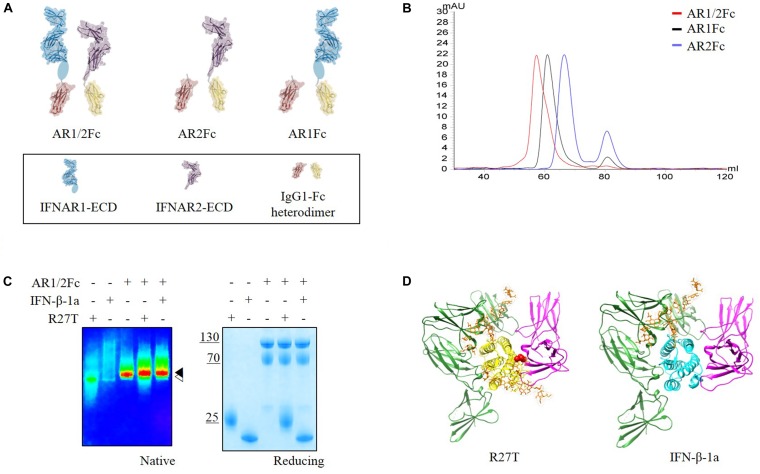
**(A)** Schematic representation of the assembly of Fc chimeric receptor proteins shown in different colors. IFNAR1-ECD (PDB 3S98, blue) and IFNAR2-ECD (PDB 1N6U, purple) were linked to mutant Fc (PDB 4X98, red and yellow) via a polypeptide linker (gray). Sub-domain 4 of IFNAR1-ECD was not visible in the crystal structure and is indicated by an oval shape. This illustration was generated using Molecular Maya. **(B)** Size exclusion chromatography elution profiles of three different receptors using a Superdex 200 16/600 PG column equilibrated in 20 mM sodium phosphate pH 7.4, 150 mM NaCl (flow rate = 0.5 mL/min) showing fractions for each experiment. **(C)** Complex formation of R27T and IFN-β-1a with AR1/2Fc determined by PAGE analysis under native and reducing conditions. A pseudo-color image of the native gel was obtained to show size differences between apo-IFNAR1/2 (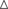) and ligand complexes (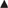). **(D)** R27T (yellow) or IFN-β-1a (cyan) was bound to the ECD of IFNAR1 (green) and IFNAR2 (magenta) in the ternary complex models, respectively. N-linked carbohydrates at N80 and N25 residues (orange) are shown as sticks. A red sphere indicates residue R27T substituted in IFN-β-1a.

Herein, we focused on interactions between IFN-βs and each receptor in the binary and ternary states. Immunoglobulin Fc heterodimer technology was employed using the previously developed ‘EW-RVT’ strategy (Choi et al., 2013) with IFNAR1-Fc, IFNAR2-Fc, and IFNAR1/2-Fc (hereafter referred to as AR1Fc, AR2Fc, and AR1/2Fc), resulting in the formation of Fc assembled proteins (Figure 1A). We compared the size of heterodimeric Fc-fusion receptors using size exclusion chromatography (Figure 1B). As expected, both R27T and IFN-β-1a formed a stable complex with AR1/2Fc, as confirmed by the presence of discrete bands in polyacrylamide gel electrophoresis (PAGE) analysis under native condition (Figure 1C). Thus, both purified monomeric AR1Fc and AR2Fc, and heterodimeric AR1/2Fc, proteins were used for ligand interactions and comparative analyses of cell-based kinetics. Furthermore, we performed *in silico* docking to elucidate the ternary complexes of R27T and IFN-β-1a with their cognate receptors, in which each receptor binds to the opposite side of the ligand (Figure 1D). As previously reported that the position of the substituted residue (R27) and the residue at which the glycosylation (N25) occurred were located in AB loop of IFN-β-1a, which is the binding interfaces of IFNAR2 (Runkel et al., 2000).

### Kinetic Evaluation of R27T and IFN-β-1a in Binary and Ternary Complex Formation

**FIGURE 2:**
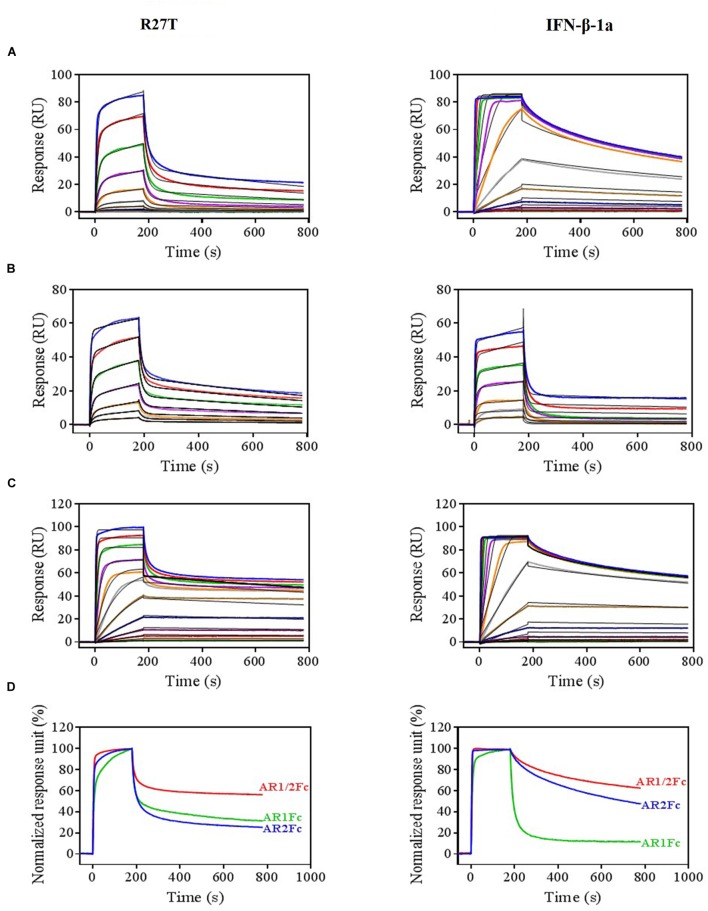
Differential kinetic behaviors of R27T and IFN-β-1a. Kinetic analysis of R27T (left panels) and IFN-β-1a (right panels) binding to AR2Fc **(A)**, AR1Fc **(B)**, and AR1/2Fc **(C)** chimeric proteins using a BIAcore T200 SPR instrument with 11 (top, 0.097–100 nM), seven (middle, 6.25–400 nM), and 12 different concentrations (bottom, 0.097–200 nM), and a CM5 gold chip with captured anti-human Fc IgG (see the section “Materials and Methods”). Association (*K*_a_) and dissociation (*K*_d_) constants were determined using the 1:1 Langmuir model (the right panel of **A**), two-state binding model (the left panel of **A**, **B**), and heterogeneous ligand model **(C)** as indicated by the black lines. **(D)** Normalized response units from sensorgrams of 100 nM R27T and IFN-β-1a with each receptor (AR2Fc, red; AR1Fc, cyan; AR1/2Fc, blue). Kinetic constants of the binding of each ligand to each receptor are listed in Tables 1, 2.

Next, we evaluated the binding kinetics of R27T and IFN-β-1a with its receptors using surface plasmon resonance (SPR) technology. It is well known that immobilization of AR1Fc, AR2Fc, and AR1/2Fc receptors through its Fc region could improve the orientation of the ligand, and hence the accessibility of the analyte. Our result showed that R27T (*K*_D_ = 16.82) has a ∼35.8-fold lower affinity compared with IFN-β-1a (*K*_D_ = 0.47) when forming binary complexes to AR2Fc (Figure 2A and Table 1). The observed binding affinity of IFN-β-1a (*K*_D_ = 0.4 nM) was similar to a previous report (Harari et al., 2014). However, the SPR sensorgrams suffered from poor fitting and did not accurately reflect the dissociation rates of R27T from AR2Fc using the sample Langmuir model (Supplementary Figure S1A), and better fitting was achieved using a two-state reaction (conformational change) model. Interestingly, the R27T-AR2Fc interaction feature shows a slow association and fast dissociation rates compared with IFN-β-1a. As we mentioned above, the N-linked glycan at the N25 residue of R27T, located at the binding interface in the receptor complex, can modulate the interaction with IFNAR2.

**Table 1:** Kinetic parameters of the interactions between IFNs (R27T and IFN-β-1a) and heterodimeric Fc-fusion receptors (AR2Fc and AR1Fc).

Ligand	Analyte	k_1a_ × 10^5^	k_1d_ × 10^-3^	*K*_D_	k_2a_ × 10^-3^	k_2d_ × 10^-3^	*K*_D_
		(M^-1^s^-1^)	(s^-1^)	(nM)	(s^-1^)	(s^-1^)	(nM)
AR2Fc	R27T^a^	9.731	62.44	-	2.739	0.9736	16.82
	IFN-β-1a^b^	75.45	3.556	0.47	-	-	-
AR1Fc	R27T^a^	4.911	83.07	-	4.161	84.46	28.54
	IFN-β-1a^a^	5.426	129.7	-	1.776	45.75	48.97


*^*a*^Sensorgrams fitted using the two-state model (A + B ↔ AB ↔ AB^∗^). ^*b*^Sensorgrams fitted using the one-to-one binding model (A + B ↔ AB).*

Because the interaction undergoes conformational changes upon ligand binding (Strunk et al., 2008; Chuartzman et al., 2017), we explored the binding kinetics of each ligand with AR1Fc using a two-state reaction model (Thomas et al., 2011). Similarly, again the sensorgrams gave a poor-fit to the Langmuir binding model (Supplementary Figures S1B,C). The results, as shown in Table 1, indicated that the value of R27T (*K*_D_ = 28.54 nM) and IFN-β-1a (*K*_D_ = 48.97 nM) with AR1Fc, revealed a clear difference in dissociation constant rates during the initial step (k_1d_ = 83.07 × 10^-3^ s^-1^ vs. 129.7 × 10^-3^ s^-1^, A + B ← AB), and association rates in the conformational change state (k_2a_ = 4.161 × 10^-3^ s^-1^ vs. 1.776 × 10^-3^ s^-1^, AB → AB^∗^) (Figure 2B). Unexpectedly, this result indicated that the complex of R27T and IFNAR1 could be formed in an efficient manner.

We next investigated the ternary complex binding of R27T- and IFN-β-1a-AR1/2Fc by fitting with heterogeneous ligand model, which resulted in R27T-AR1/2Fc (*K*_2D_ = 30.83 pM), ∼6.4-fold higher affinity compared with IFN-β-1a-AR1/2Fc (*K*_2D_ = 198.6 pM) (Figure 2C and Table 2). The binding kinetics rate constants of R27T-AR1/2Fc (1.24 × 10^5^ M^-1^s^-1^ and 1.231 × 10^-3^ s^-1^) for the first reaction revealed a similar feature of R27T-AR2Fc with a slower association and faster dissociation than IFN-β-1a-AR1/2Fc (76.85 × 10^5^ M^-1^s^-1^ and 4.435 × 10^-6^ s^-1^). In the second reaction, R27T (14.71 × 10^7^ M^-1^s^-1^ and 4.535 × 10^-3^ s^-1^) formed a more stable complex than IFN-β-1a (1.57 × 10^7^ M^-1^s^-1^ and 3.109 × 10^-3^ s^-1^). These results suggest that additional N-linked glycan of R27T in ternary complex results in rapid dissociation of IFNAR2, and enhance the interaction with IFNAR1 (Figure 2D). Therefore, we focused on the effects of the altered kinetics due to glycosylation on signal transduction and biological activity in subsequent experiments.

**Table 2:** Kinetic evaluation of the interactions between IFNs (R27T and IFN-β-1a) and heterodimeric Fc-fusion receptors (AR1/2Fc).

Ligand	Analyte	k_1a_ × 10^5^	k_1d_ × 10^-3^	*K*_1D_	k_2a_ × 10^7^	k_2d_ × 10^-3^	*K*_2D_
		(M^-1^s^-1^)	(s^-1^)	(nM)	(M^-1^s^-1^)	(s^-1^)	(pM)
AR1/2Fc	R27T^a^	1.24	1.231	9.961	14.71	4.535	30.83
	IFN-β-1a^a^	76.85	0.0044	0.0006	1.57	3.109	198.6


*^*a*^Sensorgrams fitted using the heterogeneous ligand model (A + B ↔ AB + C ↔ ABC).*

### Signal Transduction and Anti-proliferative Effects of R27T

**FIGURE 3:**
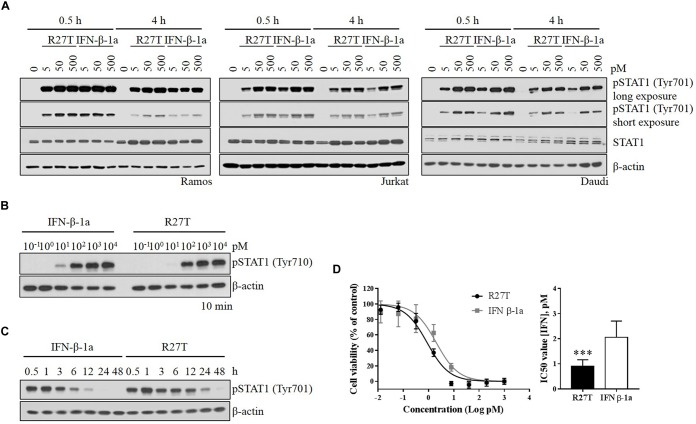
Comparison of R27T- and IFN-β-1a-induced phosphorylation of STAT1, and inhibition of cell growth. **(A)** Differential dynamics of the phosphorylation of STAT1 were verified by western blot analysis, and were time- and dose-dependent for both R27T and IFN-β-1a using various cell lines. **(B)** Daudi cells were treated with the indicated concentrations of R27T and IFN-β-1a for 10 min. **(C)** Daudi cells were treated with 5 pM R27T or IFN-β-1a for the indicated time, and phosphorylation of STAT1 was analyzed by western blotting. **(D)** Daudi cells were treated with the indicated concentrations of R27T or IFN-β-1a. After a 72 h incubation, cell viability was determined using an EZ-Cytox cell viability kit (left panel). IC50 values, the concentration at which 50% of cell growth is inhibited compared with controls, were estimated by non-linear regression using GraphPad 7.0 (right panel). IC50 values are presented as mean ± SD of three independent experiments performed in triplicate. Statistical analysis was made using student’s unpaired two-tailed *t*-test (*^∗∗∗^p* < 0.001).

To investigate the role of signaling induced by the altered binding kinetics, type I IFN receptor-expressing cells were incubated with various concentrations of R27T and IFN-β-1a for various durations. While the level of pSTAT1 peaked within 30 min upon IFN-β-1a treatment (Figure 3A, lanes 2 and 5), the response to R27T treatment lasted for 4 h (Figure 3A, lanes 9 and 12). These experiments were performed under identical conditions. Upon treatment for 10 min, the minimum concentration of IFN-β-1a required to induce production of pSTAT1 was lower than that of R27T in Daudi cells (Figure 3B). This may be because expression of the type I IFN receptor was higher on the surface of Daudi than that of other cell lines (Supplementary Figure S2). Given the observed differential induction of phosphorylation, we examined potential differences in the kinetics of phosphorylation by R27T and IFN-β-1a to measure how rapidly induction occurred, and how long it persisted, using Daudi cells. The results showed that both R27T and IFN-β-1a greatly increased the duration of pSTAT1, from 30 min to 1 h, but induction by IFN-β-1a gradually decreased over 12 h (Figure 3C, lanes 1 to 7). Interestingly, differences in the kinetics of R27T-induced pSTAT1 were not only evident after 1 h; elevated pSTAT1 persisted for up to 24 h (Figure 3C, lanes 8 to 15). In addition, the anti-proliferation potency of IFN-β in Daudi cells was evaluated, and the IC_50_ value for R27T (0.90 ± 0.26 pM) was approximately twofold lower than that for IFN-β-1a (2.06 ± 0.62 pM) (Figure 3D). Thus, these data suggest that R27T induced prolonged signal transduction with enhanced anti-proliferative activity.

### Kinetics of IFN-β Stimulated Gene Expression

**FIGURE 4:**
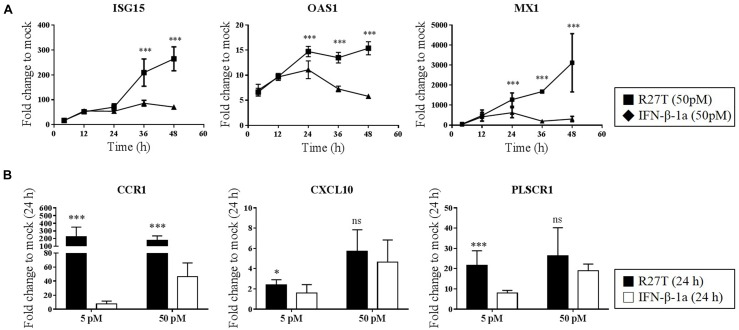
Comparison of ISG transcript following R27T or IFN-β-1a stimulation. Daudi cells were treated with the indicated concentration of R27T or IFN-β-1a. At the indicated time points, cells were harvested for mRNA isolation. **(A,B)** *ISG15, OAS1*, and *Mx1* mRNA levels at 50 pM treated samples, and expression levels of genes encoding *CCR1, CXCL10*, and *PLSCR1* with 5 or 50 pM treated samples at indicate time points determined by qRT-PCR. Data are from at least three independent experiments performed in duplicate. All results are presented as mean ± SD. ^∗^*p* < 0.05, ^∗∗∗^*p* < 0.001 as determined by one-way ANOVA, Bonferroni’s multiple comparison *post hoc* tests **(A)**, or student’s unpaired two-tailed *t*-test **(B)**.

We next identified whether the distinct biological potency and signaling kinetic properties of R27T affect the expression of ISGs. We evaluated whether the expression of type I IFN-inducible genes, including *Mx1, OAS1, ISG15, CXCL10, CCR1*, and *PLSCR1*, was upregulated by both R27T and IFN-β-1a in Daudi cells. Monitoring of IFN-sensitive genes, like *Mx1, OAS1*, and *ISG15* showed that IFN-βs upregulated gene expression in a dose-dependent manner (Figure 4A). After 24 h, the expression levels of these gene sets were significantly upregulated to a greater extent by R27T than IFN-β-1a after exposure to 50 pM. We examined the expression of the remaining three ISGs in Daudi cells treated with R27T and IFN-β-1a for 24 h in a dose-dependent manner (Figure 4B). We found that, in 5 pM concentration of R27T-treated cells the mRNA expression of *CXCL10, CCR1* and *PLSCR1* significantly up-regulated. Our results showed that R27T induced a more sustained signal transduction and gene expression than IFN-β-1a at a concentration of 5 or 50 pM. However, at a concentration of 500 pM, the effects of R27T and IFN-β-1a did not differ in both the stimulated signal transduction and increased gene expression (Supplementary Figures S3A,B). Overall, these results support a direct correlation between prolonged signal transduction and increased gene expression, following R27T treatment.

### Biophysical Differences in the Binding of R27T and IFN-β-1a to Receptors *in vitro*

**FIGURE 5:**
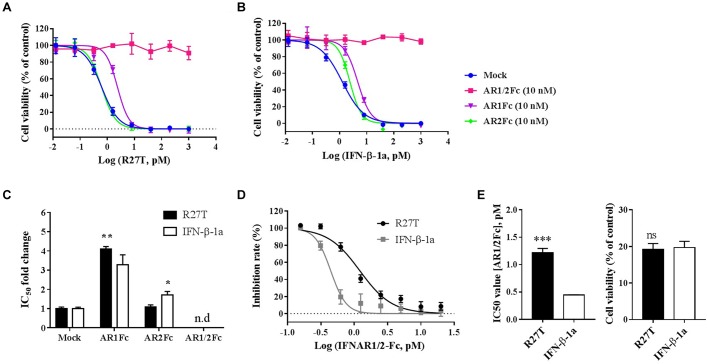
R27T and IFN-β-1a competition binding assay of Fc-fusion proteins on cells expressing type I IFN receptors. Competitive binding of R27T or IFN-β-1a with each Fc-fusion receptor protein to type I IFN receptors expressed on the cell surface was confirmed by the anti-proliferative effect on Daudi cells. Each Fc-fusion protein (10 nM) was treated with the indicated concentration of R27T **(A)** or IFN-β-1a **(B)** for 72 h, and cell viability was determined using an EZ-Cytox bioassay kit. **(C)** IC50 fold change values (normalized to mock value) of R27T or IFN-β with different Fc-fusion proteins. **(D)** Cells were exposed to the indicated concentrations of AR1/2Fc-fusion proteins with 1 nM R27T or IFN-β for 72 h. IC50 values with AR1/2Fc (The left panel of **E**) and the cell viability of each ligand without AR1/2Fc (The right panel of **E**) were determined (n.d, not detected). All experiments were repeated independently three times in duplicate **(A–C)** or triplicate **(D,E)**. Data are presented as mean ± SD. ^∗^*p* < 0.05, ^∗∗^*p* < 0.01, ^∗∗∗^*p* < 0.001 as determined by two-way ANOVA, Bonferroni’s multiple comparison *post hoc* tests **(A–C)**, or evaluated by student’s unpaired two-tailed *t*-test **(D,E)**.

To further evaluate the biophysical and biological differences of R27T and IFN-β-1a related to receptor binding on the cell surface, *in vitro* cell viability assays were performed (Figure 5). The competitive effect of each Fc-fusion receptor on the biological potency of R27T (Figure 5A) or IFN-β-1a (Figure 5B) was determined by calculating the IC_50_ fold change value, which was determined by measuring individual IC_50_ values and then dividing by control values (Figure 5C). The blocking ability of AR1Fc was more effective against R27T (4.10 ± 0.05) than IFN-β-1a (3.28 ± 0.30). In contrast, AR2Fc blocking activity was effective against IFN-β-1a (1.70 ± 0.11), but not against R27T (1.09 ± 0.06), and 10 nM AR1/2Fc completely abrogated the biological activity of both ligands. In addition, we confirmed the effective blocking of R27T or IFN-β-1a using a low concentration of AR1/2Fc (6 pM), which was more effective against R27T (15.09 ± 0.63) than IFN-β-1a (9.66 ± 0.41) (Supplementary Figure S4). These results were consistent with the *K*_D_ values obtained from SPR experiments (discussed above).

Next, we further determined the antagonistic effects using various concentrations of AR1/2Fc with a fixed concentration of ligand, and investigated how R27T and IFN-β-1a differently bind to cell surface receptors and cause biological responses (Figure 5D). As expected, the IC_50_ value for R27T (1.22 ± 0.07 nM) obtained from the competitive binding assays was found to be ∼2.7-fold higher than that for IFN-β-1a (0.44 ± 0.01 nM) (Figure 5E, left). This experiment was determined at 1 nM with similar biological activity for both R27T and IFN-β-1a (Figure 5E, right). In summary, our findings suggest that the biological activity of R27T, which is higher than IFN-β-1a, is due to differences in receptor binding kinetics on the cell surface.

## Discussion

We previously developed R27T with an additional glycosylation at residue 25 following site-directed mutagenesis of Arg27 to Thr (Song et al., 2014). This glycan is on the proposed IFNAR2-binding site, according to alanine scanning mutagenesis (Runkel et al., 2000), and this residue may be able to interact with sub-domain 1 of IFNAR2 according to the two-dimensional interaction map of the IFN-α2-IFNAR2 interface (Thomas et al., 2011). Site-directed mutagenesis and computational prediction studies suggest that R27 on the AB loop is also able to influence biological activity by affecting binding to receptors (Runkel et al., 1998b, 2000; Samoudi et al., 2015). However, this residue would not be involved in interaction with IFNAR2 because it is masked by a nearby oligosaccharide (N25). Therefore, it was not considered in this study. The additional *N*-glycosylation improved various biophysical properties including solubility, stability, productivity, and pharmacokinetics, and unexpectedly enhanced anti-proliferative effects compared with IFN-β-1a (Song et al., 2014). This improved anti-proliferative effect indicated the ligand–receptor affinity was changed (Kalie et al., 2007).

**FIGURE 6:**
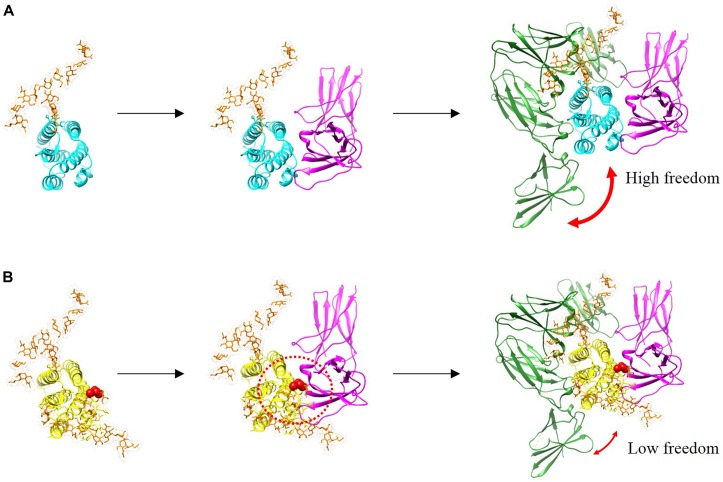
Binding complexes formed by IFN-β-1a (**A**, cyan) and R27T (**B**, yellow) to binary and ternary assemblies with the ECD of IFNAR1 (green) and IFNAR2 (magenta) are presented with modeled structures. N-linked glycan at N80 and N25 residues (orange) are shown as sticks. A red sphere indicates residue R27T substituted in IFN-β-1a. Additional glycosylation of R27T induces the steric hindrance that affects its interaction with IFNAR2, and R27T likely has less conformational freedom than IFN-β-1a in the ternary complex.

In this study, we investigated whether the additional N-linked glycan of R27T modulates receptor binding kinetics. In the case of IFN-α mutant studies, alteration in receptor interaction regulates cellular responses such as signal transduction and gene expression which explains the ability of IFN-a mutants to alter their biological activities (Piehler et al., 2012). In addition, there are such studies in which alteration in the binding dynamics of cytokine–receptor interactions can provide an understanding of functional selectivity in cytokine signaling and biological activities (Junttila et al., 2012; Moraga et al., 2014; Kim et al., 2017). Here, we presented the sequential docking structure based on the previously determined docking structures in complex with IFNAR1 and IFNAR2 (Thomas et al., 2011; De Weerd et al., 2013), to compare the different receptor-binding modes between R27T and IFN-β-1a (Figure 6). In the ternary assembly with IFNAR2 and IFNAR1, R27T reflected the kinetic feature of the binary interaction with each receptor, respectively. The glycan in R27T, locating on the binding surface for IFNAR2, induced rapid dissociation in complex formation with IFNAR2, but served for efficient binding with IFNAR1 that could involve a change in the conformational state. Thus, R27T was more efficient in forming ternary complexes but also dissociated more rapidly than IFN-β-1a. In contrast, IFN-β-1a formed stable complexes with IFNAR2 in binary and ternary assembly. This is consistent with the previous suggestion that the binding energy of the initial IFNAR2 interaction is responsible for the stability of type I IFN-receptor complex (Runkel et al., 2000). Our finding proposes that N-linked glycan on residue 25 of R27T can shield the binding interface (indicated by a dotted red circle) and induce steric hindrance, simultaneously enhance binding to IFNAR1 by restricting the conformational freedom of R27T. There are several possible explanations for such a result. Although the exact effects of the N-linked glycan on conformational change of PPIs are controversially debated, they may contribute to the changes in protein structure and biological functions. For example, an N-linked glycan on residue 80 of IFN-β-1a has been reported to stabilize the IFN-β-1a structure (Runkel et al., 1998a), and the R27T structure can be stabilized by the hydrogen bond between 35R and N25-linked glycan moieties (Song et al., 2014). It has been reported that the individual glycoforms of IFN-β-1a enable to affect the alternation in biological activities (Mastrangeli et al., 2014). In addition, the glycan enables to decrease the flexibility of the protein backbone and increase protein conformation stability (Wormald and Dwek, 1999). In the binding dynamic of PPIs, glycans cannot only contribute to steric hindrance and conformational changes (Darling et al., 2002; Bager et al., 2013; Jiang et al., 2016), but also restrict the conformational freedom of the glycosylated protein, and thereby enhance trans binding (Langer et al., 2012; Matoba et al., 2017). Therefore, it is possible that the N25-linked glycan of R27T enables to limit the flexibility of the R27T structure and thus participate in the binding kinetics with IFNAR1. A further study with more focus on the structural dynamic of R27T should be done to explain this altered receptor-binding kinetics. Also, our results showed clear differences between R27T and IFN-β-1a of receptor-binding and cellular responses.

In canonical signaling pathway, type I IFNs activate the STAT molecules upon binding on its cognate receptors on the cell surface, which in turn induce a variety of type I IFN-sensitive genes (Platanias, 2005; Schoggins and Rice, 2011; Schneider et al., 2014). In a previous study, the affected genes could be divided into robust and tunable categories (Sharma et al., 2016). The induction of robust genes is highly sensitive to the ligand-induced association of the type I IFN receptor and is related to the antiviral activity of type I IFN (Levin et al., 2014; Sharma et al., 2016). Conversely, the induction of tunable genes does not occur via ISRE sequences and is associated with anti-proliferative and immunomodulatory activities (Kalie et al., 2008; Levin et al., 2014). In this study, we determined the phosphorylation kinetics of signaling molecule, pSTAT1, by IFN-β mediated signaling pathway in the Ramos, Jurkat, and Daudi cell lines. STAT1, the primary transcription factor activated by IFN, has been studied as a more sensitive and reliable biomarker than other pathway-specific activated STATs and monitored in multiple sclerosis patients after IFN-β administration (Gavasso et al., 2014). Here, our data revealed that R27T activated STAT1 differently to IFN-β-1a and modulated the expression of IFN-inducible genes, such as robust genes (*ISG15*, *OAS1*, *Mx1*, and *PLSCR1*) and tunable genes (*CCR1* and *CXCL10*). These genes are also identified as sensitive biomarkers for IFN-β treatment in multiple sclerosis (Sellebjerg et al., 2008, 2009; Cepok et al., 2009; Hecker et al., 2011, 2013; Malhotra et al., 2011). Interestingly, we found that pSTAT1 activation was delayed by R27T compared to IFN-β-1a. IFN-β-1a appears to provide a faster opportunity for the formation of a ternary complex due to more stable binary complexes with IFNAR2 in the initial step of the binding mechanism. Nevertheless, R27T showed more sustained signal activity and increased gene expression than that of IFN-β-1a. These prolonged cellular responses by R27T occur through the canonical signaling pathway by the ligand-induced association of type I IFN receptor but not by IFNAR1-dependent alternative signaling axis, because the additional non-canonical pathway between IFN-β and IFNAR1 is independent of STAT activation (Karaghiosoff et al., 2003). We suggest that the binding kinetics of the R27T-receptor complex modulate the strength and duration of cellular activity.

In summary, we confirmed that the additional glycosylation at the N25 residue of R27T regulated receptor-binding kinetics and ternary complex formation. Consequently, the differential binding kinetics of R27T with receptors result in prolonged cellular responses and enhanced biological function compared with IFN-β-1a. Thus, we suggest that these biological and biophysical properties of R27T, like the prolonged response and protein stability, could prove to be a benefit in the treatment of multiple sclerosis. Furthermore, this approach for receptor-binding kinetics can be applied to studies for site-specific modification and conjugation of protein therapeutics.

## Author Contributions

SL, KS, and YS designed the study. SL, WS, S-SK, J-SC, and YS contributed to the conceptual development and experimental design. SL, WS, HY, and NR performed the experiments and analyzed the data. SL, SH, JC, KS, and YS prepared the manuscript.

## Conflict of Interest Statement

JC, KS, and YS currently hold stock in ABION Inc., and JC currently holds stock options in ABION Inc. The remaining authors declare that the research was conducted in the absence of any commercial or financial relationships that could be construed as a potential conflict of interest.

## Footnotes

1 http://www.glycam.com
