# Supplementary material for: A Glycoengineered Interferon-β Mutein (R27T) Generates Prolonged Signaling by an Altered Receptor-Binding Kinetics
Source: Front Pharmacol. 2019 Jan 24;9:1568. doi: 10.3389/fphar.2018.01568 (PMC6353837; doi:10.3389/fphar.2018.01568)
Supplement: Supplementary file 1 [file Data_Sheet_1.pdf]

Supplementary Material

A Glycoengineered Interferon-β Mutein (R27T) Generates Prolonged Signaling by an Altered Receptor-binding Kinetics

Saehyung Lee, Woo Sung Son, Ho Bin Yang, Nirmal Rajasekaran, Sung-Su Kim, Sungyoul Hong, Joon-Seok Choi, Jun Young Choi, Kyoung Song*, and Young Kee Shin*

*** Correspondence:** Kyoung Song: sk17@logonebio.org; Young Kee Shin: ykeeshin@snu.ac.kr

1 Supplementary Materials and Methods

Flow cytometry analysis Daudi, Jurkat, Ramos, THP-1, and MDA-MB-231 cells were used for flow cytometry analysis. Harvested cells were washed with DPBS (Hyclone Laboratories Inc., Logan, UT, USA) and placed in separate tubes at a cell density of 2.0×10^5 cells. Antibodies (anti-human IFNAR1, 85228, Thermo Fisher; anti-human IFNAR2, 10359-MM07-A, Sino Biological Inc.) were then used to determine the level of expression of type I IFN receptors in cells, and analyzed using a BD FACSCalibur instrument (BD Biosciences, Franklin Lakes, NJ, USA).

SPR analysis, western blot, real-time PCR, and competitive binding assays All experimental methods were performed as described in the Materials and Methods section.

2 Supplementary Figures and Tables

2.1 Supplementary Figures

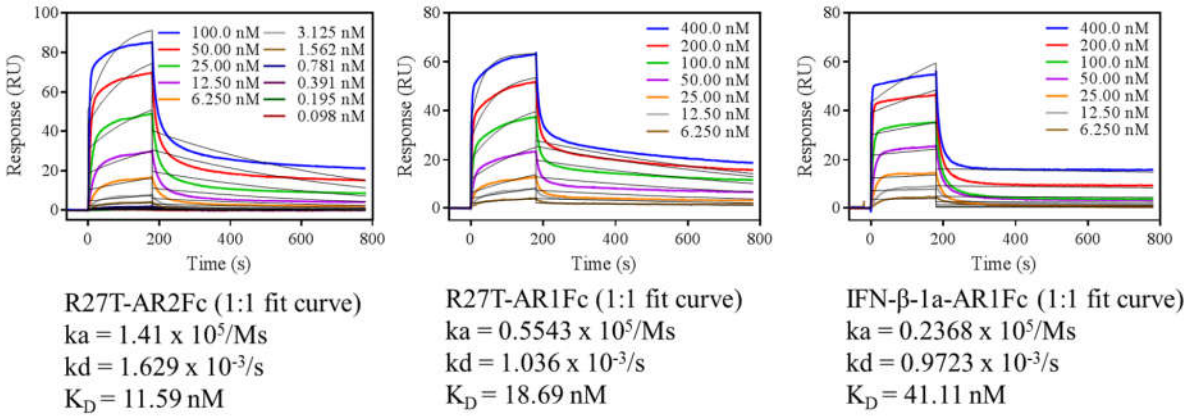

Supplementary Figure S1. Fitting of SPR data using the 1:1 Langmuir model. Kinetic analysis of R27T binding to AR2Fc (A) and AR1Fc (B), and IFN-β-1a binding to AR1Fc (C). Fusion proteins were evaluated using a BIAcore T200 instrument at 11 (A, 0.098 to 100 nM) and seven (B and C, 6.25 to 400 nM) different concentrations with a CM5 gold chip containing captured anti-human Fc IgG.

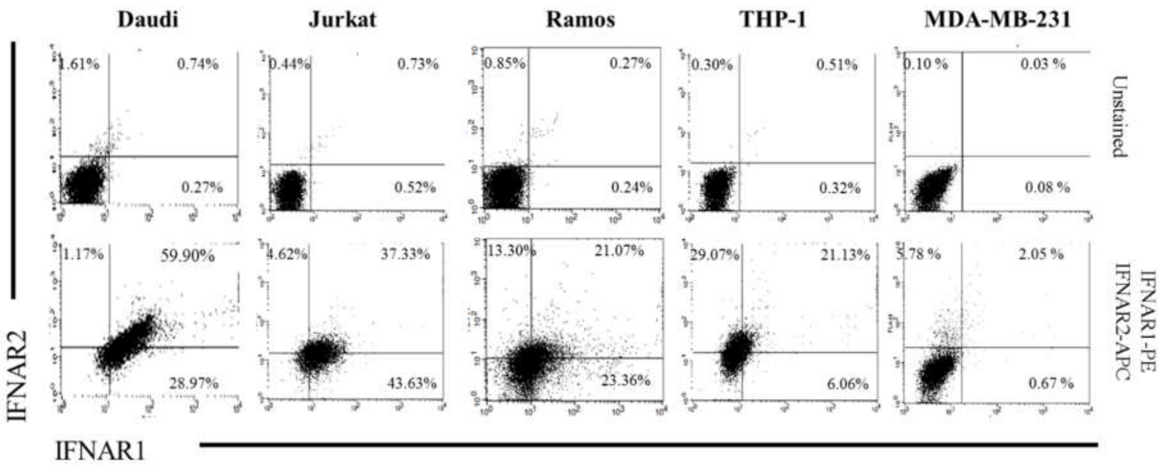

Supplementary Figure S2. Screening of the expression of type I IFN receptors on the cell surface in various cell lines by flow cytometry analysis.

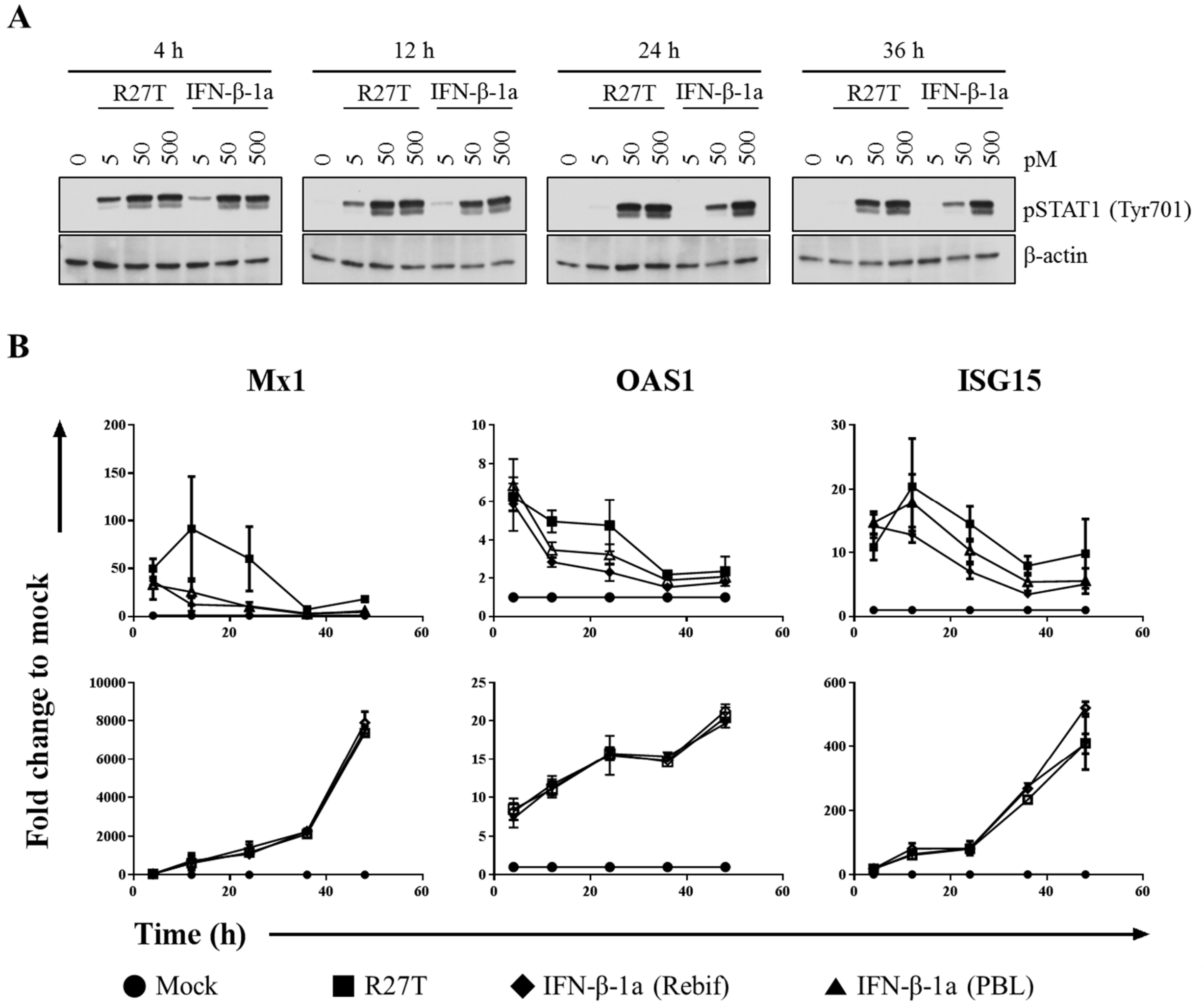

Supplementary Figure S3. (A) STAT1 phosphorylation analysis by western blotting. (B) Comparison of IFN-sensitive ISG levels following stimulation with R27T or IFN-β-1as. Mx1, OAS1, and ISG15 mRNA levels in 5 pM (upper) or 500 pM (lower) treated samples were determined at the indicated time points by qRT-PCR analysis.

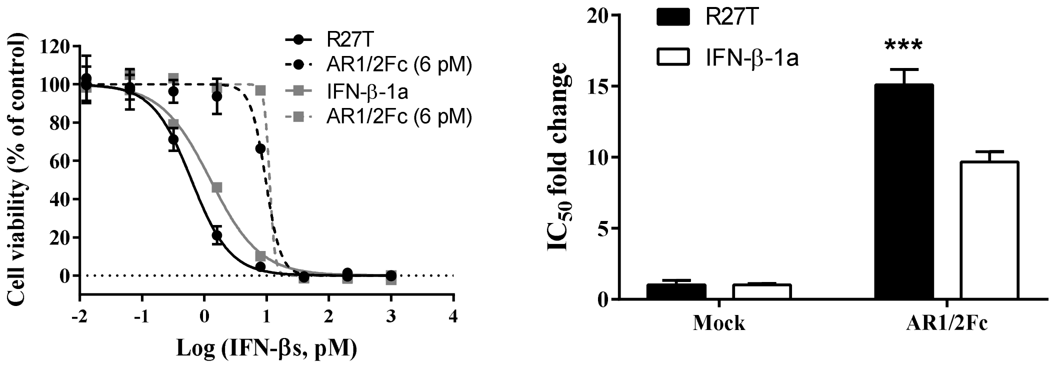

Supplementary Figure S4. Displacement of AR1/2Fc (6 pM) by increasing concentrations of R27T or IFN-β-1a on Daudi cells expressing type I IFN receptors. IC_{50} values with AR1/2Fc and cell values are means ± s.d. of three independent experiments performed in triplicate. ****p* < 0.001 (two-way ANOVA, Bonferroni's multiple comparison *post hoc* tests).

2.2 Supplementary Table

Supplementary Table 1. Oligonucleotide primers used for real-time PCR

Gene	Primer sequence	Reference
GAPDH	F: 5′-TCCCTGAGCTGAACGGGAAG-3′	(Ahmad et al., 2013)
	R: 5′-GGAGGAGTGGGTGTCGCTGT-3′	
ISG15	F: 5′-TCCTGGTGAGGAATAACAAGGG-3′	(Du et al., 2012)
	R: 5′-GTCAGCCAGAACAGGTCGTC-3′	
Mx1	F: 5′-TCCCACCCTCTATTACTGAATGG-3′	(Du et al., 2012)
	R: 5′-GGGAAGGGCAACTCCTGAC-3′	
OAS1	F: 5′-CATCCGCCTAGTCAAGCACTG-3′	(Kato et al., 2004)
	R: 5′-CCACCACCCAAGTTTCCTGTAG-3′	
CXCL10	F: 5′-TGCTGGGTCTGAGTGGGACT-3′	(Varona et al., 2005)
	R: 5′-CCCTATGGCCCTCATTCTCAC-3′	
CCR1	F: 5′-CTCTTCCTGTTCACGCTTCC-3′	(Martínez-Iglesias et al., 2016)
	R: 5′-GCCTGAAACAGCTTCCACTC-3′	
PLSCR1	F: 5′-GAATGCTTCTCACCCGGAAA -3′	(Song et al., 2011)
	R: 5′-TCCTGGAGGTCCTTGGAATG-3′	

3 Reference

Ahmad, T.a.F.T., Jubri, Z., Rajab, N.F., Rahim, K.A., Yusof, Y.a.M., and Makpol, S. (2013). Gelam honey protects against gamma-irradiation damage to antioxidant enzymes in human diploid fibroblasts. *Molecules* 18, 2200-2211.

Du, Z., Whitt, M.A., Baumann, J., Garner, J.M., Morton, C.L., Davidoff, A.M., and Pfeffer, L.M. (2012). Inhibition of type I interferon-mediated antiviral action in human glioma cells by the IKK inhibitors BMS-345541 and TPCA-1. *Journal of Interferon & Cytokine Research* 32, 368-377.

Kato, A., Ogasawara, T., Homma, T., Batchelor, J., Imai, S., Wakiguchi, H., Saito, H., and Matsumoto, K. (2004). CpG oligodeoxynucleotides directly induce CXCR3 chemokines in human B cells. *Biochemical and biophysical research communications* 320, 1139-1147.

Katoh, H., Hosono, K., Ito, Y., Suzuki, T., Ogawa, Y., Kubo, H., Kamata, H., Mishima, T., Tamaki, H., and Sakagami, H. (2010). COX-2 and prostaglandin EP3/EP4 signaling regulate the tumor stromal proangiogenic microenvironment via CXCL12-CXCR4 chemokine systems. *The American journal of pathology* 176, 1469-1483.

Martínez-Iglesias, O.A., Alonso-Merino, E., Gómez-Rey, S., Velasco-Martín, J.P., Orozco, R.M., Luengo, E., Martín, R.G., De Cáceres, I.I., Fernández, A.F., and Fraga, M.F. (2016). Autoregulatory loop of nuclear corepressor 1 expression controls invasion, tumor growth, and metastasis. *Proceedings of the National Academy of Sciences* 113, E328-E337.

Song, G., Fleming, J.-a.G., Kim, J., Spencer, T.E., and Bazer, F.W. (2011). Pregnancy and interferon τ regulate DDX58 and PLSCR1 in the ovine uterus during the peri-implantation period. *Reproduction* 141, 127-138.

Varona, R., Cadenas, V., Gómez, L., Martínez-A, C., and Márquez, G. (2005). CCR6 regulates CD4+ T-cell–mediated acute graft-versus-host disease responses. *Blood* 106, 18-26.
